# Determinants of Higher-Risk Sexual Behavior in Some Selected African Countries

**DOI:** 10.1155/2021/8089293

**Published:** 2021-09-03

**Authors:** Adikwor Ewoenam Puplampu, Seth Afagbedzi, Samuel Dery, Dzifa Adimle Puplampu, Chris Guure

**Affiliations:** ^1^Department of Biostatistics, School of Public Health, University of Ghana, Legon, Accra, Ghana; ^2^Center for Climate Change and Sustainable Studies, University of Ghana, Legon, Accra, Ghana

## Abstract

**Background:**

Although higher-risk sexual behavior (H-RSB) is a major contributor to the rapid rising rate of new HIV infections, there exists paucity of comprehensive evidence across the sub-Saharan African region. The purpose of this study was to determine the prevalence of H-RSB and its determinants across sub-Saharan Africa to inform policy.

**Method:**

Data were obtained from the Demographic and Health Survey (DHS) of ten sub-Saharan African (SSA) countries with their three most current DHS surveys from 2000 to 2016. Only participants who ever had sexual encounters in their lifetime were included in the study. Weighted adjusted Cox regression with robust variance and constant time was used to investigate disparities of H-RSB among the ten SSA countries. Relationships between sociodemographic, socioeconomic, knowledge, mass media, and H-RSB were investigated.

**Results:**

The trend and prevalence of higher-risk sexual behavior show that Lesotho experienced a decreasing trend of the prevalence of H-RSB from 8.92 in period one to 6.42 in period three. Ghana experienced a marginal increase from 6.22 in period one to 6.76 in period two and then to 6.43 in the third period. However, Malawi, Zambia, and Zimbabwe obtained a marginal increasing trend in the prevalence of H-RSB from period one to three: 2.75 to 3.74, 4.33 to 6.24, and 6.11 to 7.99, respectively. Meanwhile, the prevalence of H-RSB in Namibia and Uganda decreased in period two to 1.84 and 5.76 but increased in period three to 2.01 and 6.83, respectively. Generally, determinants of H-RSB among the countries include age, sex, religious affiliation, marital status, educational level, employment status, economic status, age at first sex, and status of circumcision.

**Conclusion:**

Trend of relatively high prevalence of H-RSB has been found across majority of the countries with key sociodemographic factors influencing H-RSB. Therefore, different targeted interventional approaches are needed in all the countries to help reduce H-RSB and the overall HIV incidence. If issues regarding sexual behavior and sexual health are not addressed adequately, H-RSB can negate all the appreciable efforts aimed at ending the HIV pandemic by 2030.

## 1. Introduction

Sub-Saharan Africa is home to an estimated 12% of the worldwide population yet accounts for more than 70% of the global burden of HIV infections [1, 2]. The adult prevalence of HIV in the region is high compared to other regions of the globe. In 2014, 70% of the infections in the world and 66% of new infections and deaths related to HIV each occurred in the subregion [1]. Again in 2017, an estimated 71% of the world's infections, 65% of new infections, and 75% of deaths related to AIDS were recorded in the region [3]. There exists a significant difference in the severity of the epidemic within the subregion [4]. There is also an association between geographical distributions and the spread of the HIV epidemic in some parts of the region [5]. Although most of the countries in the region share a considerable number of the epidemiologic attributes, substantial subregional variation exists in the severity of these epidemics [6]. Adult HIV prevalence is generally highest (>10%) in Southern Africa, intermediate in Central and East Africa, and lower (<5%) in West Africa. The trends in new HIV infections across countries in SSA have shown a decline by more than 33% from an estimated 2.2 million in 2005 to 1.5 million in 2013, but remain high [7].

In Ghana, the estimated adult HIV prevalence is 1.7%, which is low compared to other countries in the region. The HIV prevalence has remained consistently high in some African countries, notably, Swaziland 27.4%, Lesotho 23.8%, Botswana 22.8%, South Africa 18.8%, Zimbabwe 13.3%, Mozambique 12.5%, Namibia 12.1%, Zambia 11.5%, Malawi 9.6%, Equatorial Guinea 6.5%, Uganda 5.9%, Kenya 4.8%, and Tanzania 4.5% [8, 9].

According to Dixon et al. [10], the burden of the HIV epidemic caused a decline in economic growth rates in Africa by 2–4%. This has led low-income countries further into poverty [11, 12]. Households are feeling the impact of AIDS in terms of earnings and expenditure on medical care. The demands for healthcare in most countries have increased which has put a burden on their already weak health systems [13]. This situation generates several policy concerns since the socioeconomic development at household, community, and national levels are affected.

Some studies have found H-RSB as a major factor which contributes to the rapid rising rate of new HIV infections [6, 14]. This could suggest that the people of high HIV prevalence countries indulge in H-RSB and that may be responsible for the high HIV prevalence. On the contrary, other studies found that the sexual behavior of the countries with high HIV prevalence is not different from countries with low HIV prevalence [15]. It is, therefore, very important to use existing data to establish the relationship among high HIV prevalence, H-RSB prevalence, and determinants of H-RSB.

Several studies have been conducted in this area but have mostly focused on factors that cause or are associated with HIV and not necessarily H-RSB. This study seeks to fill this gap using available Demographic and Health Survey data from 10 sub-Saharan African countries. The purpose of this study was to determine the prevalence of H-RSB and its determinants across sub-Saharan Africa to inform policy.

## 2. Methods

### 2.1. Study Design and Participants

Secondary data from DHS were used in this study for sub-Sahara African countries with the three most current DHS survey from 2000 to 2016. These DHS surveys are nationally representative. Demographic and Health Survey uses the stratified two-stage sampling design approach, by drawing enumeration areas from the most current census data.

In Ghana, Kenya, Lesotho, Nigeria, Zambia, and Rwanda, men within the age group 15–59 and women within the age group 15–49 who were occupants of a household prior to the survey were eligible to participate in the interview. In Namibia, men and women between the ages of 15 and 64 were qualified for the survey for period three; however, in the first two periods, women and men within the age group 15–49 and 15–59, respectively, were included in the study. Uganda, Malawi, and Zimbabwe included men aged 15–54 and 15–49 for women in the study. Also, in Nigeria participants who qualified for the study in period three were between the ages of 15 and 49 for both men and women. DHS ensures that the sample being interviewed is representative at all levels of the national population of the respective countries.

The three different Demographic and Health Surveys for each country were grouped into three periods. The first survey for each country out of the three survey years were grouped as period one, the second survey year was grouped as period two, and the third or most recent survey year, period three as provided in Table 1.

### 2.2. Study Area

According to the World Bank Group [16], SSA is made up of 48 countries in Africa. However, for the purpose of this study, ten sub-Saharan African countries were selected to be included in the study. We selected countries with high HIV prevalence and have DHS data for continuous three periods. The ten countries selected are presented in Table 1, with their 2017 HIV prevalence [8, 17] indicated.

### 2.3. Sample Size

The total observations realized for all survey years for the ten countries were 568,779. However, since the study is about sexual behavior, only individuals who had engaged in this practice were eligible (391,132) to be part of the study.

### 2.4. Statistical Analysis

In all the analyses, we adjusted for the complex nature of the survey design by accounting for clustering, stratification, and weighting. Analysis was performed on the pooled dataset for the individual countries using Stata version 14. Frequencies and percentages were reported for categorical and dichotomous variables. Multivariate Cox proportional hazards model with robust variance estimates and constant time at risk was used to find the statistical association between H-RSB and the independent variables for each of the countries. This was performed to determine the adjusted hazard rates with their confidence intervals for the pooled dataset within each country. The multivariate Cox proportional hazard model with robust variance estimates and constant time is a powerful statistical modelling approach that allows the incorporation of explanatory variables at different levels of the hierarchy. Scholars such as Campbell, Skov et al., and Barros and Hirakata [19–21] suggest Cox regression with constant time to be an alternative to the logistic regression model. They confirmed that by declaring a constant follow-up time, the Cox regression model can be fitted for the estimation of hazard rates in cross-sectional studies notably when robust variance estimates are used. Robust variance provides better estimates in the analysis of cross-sectional studies with the binary outcome. Inclusion of variables were based on their significance or relevance to the outcome at an alpha level of 5%.

### 2.5. Outcome Variable

In this study, H-RSB is defined as having multiple sexual partners and not using condoms consistently [22–24]. The variable, multiple sexual partners were created to differentiate between individuals who had 2+ sexual partners including their wives and those who did not have. In respondents who did not have multiple sexual partners, a code of “0” was given, and in respondents who had multiple sexual partners, a code of “1” was assigned. However, for inconsistent condom use, respondents who responded “yes” to condom use at last sex and not with the wife, a code of “1” was given, and those who responded “no,” a code of “0” was assigned. A respondent was said to be practicing H-RSB if the individual responded “yes” to having multiple sexual partners and “no” to who did not use condom at last sex and not with wife; otherwise, the person is not practicing H-RSB.

## 3. Results

### 3.1. Distribution of H-RSB among the Ten Sub-Saharan Africa Countries

From the pooled dataset for each of the countries, the mean age of respondents in Ghana who practiced H-RSB was 35 years. Ghanaians who were exposed to watching television, listening to radio, reading newspaper/magazine, and had access to internet practiced H-RSB more. In Kenyan, Lesotho, Malawian, Namibian, and Nigerian participants who practiced H-RSB had an average age of 33, 32, 32, 31, and 35 years, respectively. On the average, 36, 32, and 33 years of Rwandans, Ugandans, and Zambians practiced H-RSB, respectively (Table 2).

### 3.2. Trend and Prevalence of H-RSB in the Ten SSA Countries

**Figure 1:**
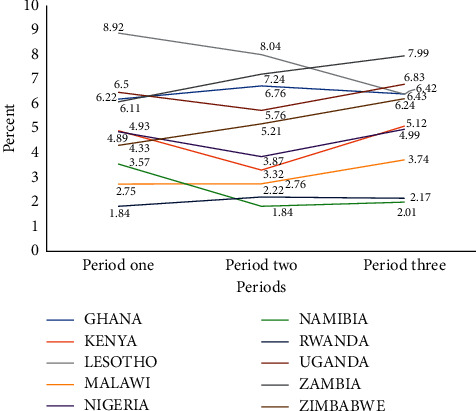
Trend of higher-risk sexual behavior across periods for each country.

The trend of the prevalence of higher-risk sexual behavior highlights that Lesotho experienced a decreasing trend of H-RSB from 8.92 in period one to 6.42 in period three. Ghana experienced a marginal increase from 6.22 in period one to 6.76 in period two and it decreased to 6.43 in the third period. However, Malawi, Zambia, and Zimbabwe obtained a marginal increasing trend in the prevalence of H-RSB from period one to three. Meanwhile, the prevalence of H-RSB in Namibia and Uganda decreased in period two to 1.84 and 5.76 but increased in period three to 2.01 and 6.83, respectively. Rwanda also experienced a marginal increase in the prevalence of H-RSB from period one to two and decreased marginally in the third period. Kenya and Nigeria also experienced a decline in the prevalence of H-RSB from period one to two and then increased in the third period (Figure 1).

### 3.3. Bivariate Analysis of H-RSB with Country

Table 3 presents the result of the Cox regression model with constant time for examining whether there is an association between H-RSB and each of the ten SSA countries. Comparing all the other nine countries to Rwanda, there was a significant increase hazard rate between H-RSB and the other countries at *p* < 0.001. Although all the other countries recorded a significant increase hazard rate of H-RSB, Zambians were more likely to engage in H-RSB. The risk of practicing H-RSB if you are a Zambian is 4.66 times significantly higher compared to a Rwandan (HR: 4.66 at 95% CI 4.26, 5.09; *p* < 0.001). Subsequently, the risk of H-RSB in Lesotho is 4.26 times compared to Rwanda (HR: 4.26 at 95% CI 3.86, 4.69; *p* < 0.001). Meanwhile, Namibians were found to be 37% more likely to indulge in H-RSB compared to Rwandans (HR: 1.37 at 95% CI 1.22, 1.55; *p* < 0.001).

### 3.4. Multivariate Cox Regression Analysis of Higher-Risk Sexual Behavior

A Cox regression model with constant time was performed to obtain the effect of factors on H-RSB from the pooled dataset for each country.  Table 4 presents the adjusted hazard ratios and their 95% confidence intervals with their corresponding *p* values for each of the countries. In Ghana, individuals whose first sex was before 18 years were 74% more likely to engage in H-RSB compared to those after 18 years (aHR: 1.74 at 95% CI 1.47, 2.06; *p* < 0.001). Respondents in the age groups 20–24 years had a 54% lower risk of practicing H-RSB compared to the age group (15–19) years (aHR: 0.46 at 95% CI 0.22, 1.00; *p* < 0.05). Also, respondents cohabiting was 46% more likely to engage in H-RSB than those married (aHR: 1.46 at 95% CI 1.16, 1.83; *p* < 0.01). However, females had a 99% lower risk of indulging in H-RSB than males (aHR: 0.01 at 95% CI 0.00, 0.04; *p* < 0.01).

In Kenya, respondents whose first sex was before 18 years were more than two times likely to practice H-RSB (aHR: 2.15 at 95% CI 1.04, 2.50; *p* < 0.001). Circumcised men were 28% less likely to engage in H-RSB (aHR: 0.72 at 95% CI 0.58, 0.91; *p* < 0.01). With the exception of divorcees, all other levels of marital status were more likely to engage in H-RSB than individuals who had never married. Moreover, females in Kenya were 79% less likely to indulge in H-RSB compared to males.

The study found that individuals whose first sex was before 18 years were 50% more likely to practice H-RSB in Lesotho (aHR: 1.50 at 95% CI 1.31, 1.72; *p* < 0.01). Also, richest, richer, and middle-class respondents were 38%, 36%, and 29% less likely to indulge in H-RSB, respectively. Spouses or partners who lived together had a 21% low risk of engaging in H-RSB (aHR: 0.79 at 95% CI 0.69, 0.90; *p* < 0.01) compared to those who did not live together. Moreover, females were 58% less likely to practice H-RSB in Lesotho.

Malawians whose first sex was before 18 years were 46% more likely to engage in H-RSB (aHR: 1.46 at 95% CI 1.27, 1.68; *p* < 0.001). However, a higher age was associated with a reduced hazard rate of practicing H-RSB. Also, females had 96% low risk of indulging in H-RSB. Again, Islamists and individuals who did not belong to any religion were 41% and 59% more likely to engage in H-RSB, respectively, compared to Christians.

Individuals whose first sex was before 18 years recorded a 99% hazard rate of H-RSB in Namibia (aHR: 1.99 at 95% CI 1.62, 2.44; *p* < 0.001). Females were 80% less likely to engage in H-RSB. Respondents who were separated recorded the highest hazard rate of H-RSB (HR: 2.13 at 95% CI 1.36, 3.34; *p* < 0.01), followed by cohabiting partners (aHR: 1.73 at 95% CI 1.31, 2.29; *p* < 0.001) compared to individuals who had never married.

Nigerians whose first sex was before 18 years were 55% more likely to engage in H-RSB. Although respondents who knew their HIV status were 14% less likely to indulge in H-RSB, respondents who had heard of AIDS were 35% more likely to engage in H-RSB. Spouses or partners who reside together had 19% high risk of practicing H-RSB (aHR: 1.19 at 95% CI 1.01, 1.41; *p* < 0.05).

In Rwanda, respondents in the age group (20–24) were 68% less likely to indulge in H-RSB than the age group (15–19) (aHR: 0.32 at 95% CI 0.10, 0.96; *p* < 0.05). Females had 94% low risk of engaging in H-RSB. Individuals in poorer class recorded a 42% increase hazard rate of practicing H-RSB and Rwandans who did not belong to any religion were more than two times likely to engage in H-RSB compared to Christians.

Islamist in Uganda were 46% more likely to engage in H-RSB compared to Christians. Urban area residents were 17% less likely to practice H-RSB (aHR: 0.83 at 95% CI 0.71, 0.97; *p* < 0.05). Ugandans whose first sex was before 18 years were 52% more likely to practice H-RSB (aHR: 1.52 at 95% CI 1.38, 1.67; *p* < 0.01).

Individuals whose first sex was before 18 years were at 48% high risk of practicing H-RSB in Zambia. Respondents in urban areas were 33% less likely to indulge in H-RSB (aHR: 0.67 at 95% CI 0.59, 0.77; *p* < 0.01) and females were 94% less likely to engage in H-RSB.

In Zimbabwe, the results show that females were 96% at low risk of practicing H-RSB. First sex before 18 years recorded 80% increased hazard rate of practicing H-RSB. Zimbabweans who knew their HIV status were at 15% low risk of engaging in H-RSB (Table 4).

## 4. Discussion

Despite the decreasing prevalence of HIV in SSA, the proportion of H-RSB among Malawians, Zambians, and Zimbabweans consistently increased across all survey years. Meanwhile in Ghana, Rwanda, Nigeria, Uganda, Kenya, and Namibia, the proportions of H-RSB in these countries are pendulum-like across the survey years. However, the proportion of H-RSB in Lesotho kept decreasing over the survey years. Lesotho is seeing a reduction in the prevalence of H-RSB because of the improvement in BCC programs aimed at preventing HIV in the country [25]. However, countries experiencing pendulum-like trend of H-RSB may be because the interventions aimed at targeting BCC programs have not seen any much improvement. Also, the unavailability of funds from donor countries has caused campaigns aimed at promoting safer sexual behaviors including modes of HIV prevention to stall [26]. There is a higher prevalence of H-RSB in Zimbabwe. This may be attributed to underfunded HIV prevention programs in Zimbabwe [26]. Also, a study by Meekers in Zimbabwe revealed that most men in Zimbabwe despite their knowledge in condom use and its benefits, do not use it during sexual intercourse [27].

The results further showed that respondent who had “first sex” at early age consistently recorded a significant increase in the hazards of engaging in H-RSB across all countries. This is in line with studies by Marston et al. [28] which observed that sexual intercourse at an early age meant there was a high probability of having more lifetime partners and practicing H-RSB which increased the risk of new infection. Consistently across all countries, females recorded a significant reduced risk of practicing H-RSB. Across most of the countries, participants within the last levels of age groups comprising 50–54, 55–59, and 60–64 recorded the highest proportion of practicing H-RSB. This is agreeable with a study by Uchudi et al., which indicate that older men may have more money and can afford many sexual partners. These groups of people do not use condoms consistently and may practice H-RSB [29].

The study revealed that the proportion of workers who engaged in H-RSB were more than nonworkers in all the countries. In Kenya, Lesotho, Namibia, and Zimbabwe, workers contributed to significantly increasing H-RSB than nonworkers. Workers may be exposed to wealth and may be at a greater advantage of engaging in transactional sex which may result in increasing the number of individuals practicing H-RSB [30, 31]. Findings from this study indicate that across all countries, males practice H-RSB more than females. Alsan Cutler and Silas [32, 33] revealed that when the earnings of males' increase, there is an increase in H-RSB due to the high-income effect. Bingenheimer [4] also found an association between male and multiple sexual partners which contributes significantly to the spread of the HIV epidemic in SSA. Gillespie et al. [34] revealed that even when women are financially sound, they are still at risk of HIV infection due to the extra economic benefits of engaging in unsafe sexual practices.

Respondents who were married or cohabiting recorded a high proportion of H-RSB in most of the countries. Married couples or cohabiting couples may not use condoms consistently because of their desire to give birth; meanwhile, their partners may have other partners which will put them at risk of new infections. This is in line with a study which observes that couples who are in lengthier and more stable relationships may desire to have children, thereby, resulting in inconsistent condom use and putting them at risk of the infection [35]. With the exception of Nigeria and Zambia, most of the respondents did not know their HIV status or had never tested for HIV before which may put them and their partners at risk of getting infected with the virus.

Although respondents who read newspaper or magazine had the increased risk of practicing H-RSB in Zambia and Zimbabwe, the reverse was the case in Namibia. As emphasized by Uchudi et al. [29], being exposed to mass media (listening to radio, reading newspaper, and watching television) in places where majority of the people are illiterates or semiliterates can help reduce people's involvement in H-RSB by informally treating and explaining topics related to sexual health, sexual behavior, and HIV prevention. However, Klein et al. [36] are also of the view that when people are exposed to watching television, reading newspapers or magazines, and listening to radio, they engage more in H-RSB. They further explained that the choice of programs or videos they are exposed to may contribute more to their engagement in H-RSB. Lesotho, Namibia, and Rwanda had majority of their respondents not having comprehensive knowledge of HIV/AIDS; this could be attributed to the fact that most of their respondents attained only primary education (Lesotho and Rwanda) and no education (Namibia). With the exception of Kenya, Rwanda, and Zimbabwe, most of the respondents of H-RSB lived in the rural areas.

Residing in urban areas influenced H-RSB positively in Nigeria, Uganda, and Zambia. Kayeyi et al. [37] is of the view that people who live in urban areas are highly likely to have comprehensive knowledge about HIV because they have been largely exposed to mass media and the availability of resources (access to condoms). These encourage them to desist from activities that will increase the risk of infection.

## 5. Conclusion

Despite the declining prevalence of HIV in sub-Saharan Africa, higher-risk sexual behavior marginally continues to increase in most of the countries. Higher-risk sexual behavior continues to be a paramount factor that contributes significantly to the risk of new HIV infections in SSA. The findings from this study highlights that there are disparities in H-RSB across the various countries.

Zimbabwe recorded an increasing trend in H-RSB, and there exists a significant variation in the increasing trend of H-RSB. Lesotho, however, recorded a decreasing trend in H-RSB, and there was a significant variation in the decreasing trend. Whereas, most of the other countries had a pendulum-like trend in H-RSB, and there were no significant variations. Eventhough more women live with HIV, more men practice H-RSB in the subregion.

Unlike the other determinants, age at first sex consistently contributes significantly to increasing H-RSB in all ten countries. Therefore, in order for the achievement of SDG 3 target 3 to be a reality, reduction in risky sexual behavior should be taken a critical look at in SSA.

### 5.1. Strengths, Implications, and Limitations of This Study

Demographic and Health Survey is one of the most relied upon data in sub-Saharan Africa in estimating and projecting individual, community, and country level indicators. Data from all DHS participating countries are standardized and therefore follow a similar multistage sampling approach. This allows for within country and between country comparisons of indicators. Therefore, estimates provided in this study are accurate and reliable. Our results provide a clear evidence on how behavior change communication (BCC) programs are to be channeled in order to help end the HIV epidemic by 2030 as stipulated by the UNAIDS. In addition to the UNAIDS fast target aimed at ending the epidemic by 2030, there is a clear evidence that sexual behavior is a major contributor to the HIV epidemic, so in addition to diagnosing and treating the disease, it should be taken a critical look at. The findings highlight the importance of education in reducing the practice of H-RSB. The findings also highlight the point that comprehensive knowledge about HIV in most of the countries did not have a positive impact on H-RSB. In most countries where a significant number of individuals knew their HIV status, they refrained from practicing H-RSB. Therefore, it is prudent for every individual to know his/her HIV status to deter them from indulging in H-RSB. Exposure to mass media had a negative influence on H-RSB; therefore, policy makers should regulate the things people watch on television and others. Some countries with high HIV prevalence in SSA were not included in the study because of unavailability of data from DHS. Due to recall bias and sensitivity of questions on sexual behavior, there were quite a number of missing observations in the dataset, and this could have affected the overall results since our analyses used a complete case approach.

## Figures and Tables

**Table 1:** Selected countries and their 3 most recent survey years.

Country	HIV prevalence (2017)	Period one	Period two	Period three
Ghana	1.7	2003	2008	2014
Kenya	4.8	2003	2008/09	2014
Lesotho	23.8	2004	2009	2014
Malawi	9.6	2004	2010	2015/16
Namibia	12.1	2000	2006/07	2013
Nigeria	2.8	2003	2008	2013
Rwanda	2.7	2005	2010	2014/15
Uganda	5.9	2006	2011	2016
Zambia	11.5	2001/02	2007	2014
Zimbabwe	13.3	2005/06	2010/11	2015

Source: [8, 17, 18].

**Table 2:** Frequency and percentage distribution of respondents practicing higher-risk sexual behavior from the country-specific pooled dataset (periods 1–3).

Country	Ghana	Kenya	Lesotho	Malawi	Namibia	Nigeria	Rwanda
Age (mean ± SD)	35.08 (10.90)	32.63 (9.68)	32.00 (9.47)	31.70 (9.23)	30.66 (10.12)	35.45 (9.68)	35.77 (10.53)

Age group							
15–19	97 (5.93)	130 (4.50)	131 (5.24)	203 (3.06)	68 (2.20)	150 (2.00)	18 (2.02)
20–24	186 (5.14)	263 (3.80)	264 (6.14)	295 (2.28)	138 (2.55)	410 (2.90)	73 (1.57)
25–29	259 (5.97)	313 (4.06)	279 (7.44)	310 (2.63)	129 (2.57)	571 (3.30)	126 (1.81)
30–34	229 (6.00)	316 (4.91)	304 (9.86)	355 (3.74)	98 (2.23)	575 (4.06)	137 (2.12)
35–39	214 (6.13)	272 (5.36)	211 (8.62)	252 (3.38)	64 (1.92)	653 (5.30)	78 (1.66)
40–44	179 (6.40)	182 (4.57)	172 (8.67)	189 (3.67)	57 (2.08)	627 (6.61)	72 (1.94)
45–49	143 (6.28)	134 (4.79)	141 (8.59)	155 (3.95)	36 (1.93)	604 (7.31)	77 (2.64)
50–54	116 (14.89)	111 (10.5)	26 (10.10)	82 (10.44)	18 (7.06)	121 (12.47)	51 (5.51)
55–59	78 (15.54)		22 (10.11)		8 (4.22)	110 (15.59)	36 (5.75)

Sex							
Male	1316 (13.85)	1406 (9.57)	607 (12.81)	1425 (10.46)	426 (5.06)	3157 (13.3)	531 (5.12)
Female	184 (1.34)	315 (1.42)	943 (6.11)	415 (0.93)	197 (1.09)	665 (1.09)	136 (0.64)
Religion							
Christian	929 (6.03)	1445 (4.36)	1483 (7.57)	1443 (2.9)	488 (2.47)	3597 (4.54)	617 (2.02)
Islam	211 (6.1)	140 (5.96)	1 (1.33)	312 (4.19)	Na	188 (3.74)	23 (3.58)
Traditional	194 (7.31)	Na	Na	Na	Na	7 (8.43)	8 (4.32)
No religion	31 (7.45)	125 (10.86)	49 (14.69)	77 (10.16)	68 (1.45)	Na	16 (7.08)
Others	56 (10.02)	0 (0.00)	16 (8.76)	2 (4.36)	4 (4.68)	0 (0)	4 (3.20)

Marital status							
Never married	330 (7.17)	310 (4.76)	284 (5.92)	251 (4.52)	282 (2.22)	729 (6.03)	73 (2.90)
Married	883 (6.23)	1199 (4.61)	1073 (8.26)	1358 (3.07)	150 (2.14)	2875 (4.16)	276 (1.49)
Cohabiting	185 (5.98)	69 (3.68)	27 (12.53)	117 (2.69)	143 (2.67)	93 (4.94)	257 (2.79)
Widowed	9 (5.04)	23 (4.31)	86 (7.24)	18 (2.96)	2 (0.81)	24 (3.91)	8 (2.09)
Divorced	35 (7.70)	21 (4.75)	17 (8.66)	52 (3.17)	6 (3.37)	32 (6.01)	32 (6.83)
Separated	58 (8.07)	99 (6.71)	63 (7.93)	44 (2.52)	39 (4.64)	69 (10.12)	22 (3.25)

Education							
No education	218 (4.59)	122 (4.5)	122 (14.64)	182 (2.08)	99 (4.48)	1107 (3.46)	112 (1.81)
Primary	225 (5.74)	943 (4.81)	910 (9.18)	1222 (3.34)	189 (2.83)	881 (5.29)	483 (2.24)
Secondary	921 (7.12)	460 (4.43)	454 (5.58)	387 (3.41)	287 (1.87)	1407 (5.20)	62 (1.83)
Higher	137 (8.34)	197 (4.73)	64 (4.89)	48 (3.34)	47 (2.21)	428 (4.65)	10 (1.53)

Employment status							
Not working	129 (4.10)	185 (2.12)	645 (6.21)	321 (1.67)	260 (2.08)	464 (2.01)	85 (1.48)
Working	1371 (6.83)	1533 (5.45)	904 (9.23)	1518 (3.91)	361 (2.63)	3349 (5.46)	581 (2.24)

Wealth index							
Poorest	223 (6.03)	298 (5.4)	306 (10.73)	305 (2.99)	62 (2.16)	819 (4.85)	110 (1.85)
Poorer	238 (5.67)	280 (4.4)	333 (9.66)	382 (3.29)	72 (2.31)	783 (4.78)	155 (2.48)
Middle	315 (6.79)	296 (4.31)	293 (7.87)	390 (3.31)	77 (1.94)	693 (4.51)	144 (2.23)
Richer	345 (6.64)	380 (4.64)	306 (6.43)	388 (3.35)	86 (1.79)	761 (4.51)	123 (1.90)
Richest	380 (6.89)	466 (4.7)	312 (5.77)	375 (2.9)	75 (1.65)	765 (3.96)	135 (2.04)

Residence							
Rural	779 (6.48)	1036 (4.40)	1187 (8.58)	1558 (3.27)	314 (2.52)	2623 (4.89)	554 (2.09)
Urban	721 (6.43)	685 (5.16)	363 (5.72)	281 (2.71)	309 (2.22)	1199 (3.84)	114 (2.17)

Resides with spouse/partner							
No	139 (4.59)	198 (3.74)	463 (8.81)	80 (1.75)	41 (1.90)	164 (2.80)	24 (1.33)
Yes	626 (5.21)	900 (4.24)	644 (8.00)	1195 (2.86)	170 (1.76)	2608 (4.12)	395 (1.69)

First sex							
Above 18 years	264 (5.88)	504 (2.93)	743 (6.75)	749 (2.78)	230 (1.66)	2677 (4.35)	474 (1.72)
Below 18 years	180 (6.80)	1217 (6.19)	792 (8.80)	1089 (3.5)	388 (3.14)	1134 (4.91)	194 (4.55)

Circumcision							
No	Na	115 (12.71)	156 (9.81)	816 (9.99)	172 (3.90)	20 (12.86)	329 (5.32)
Yes	Na	993 (9)	455 (14.32)	366 (12.73)	77 (4.72)	2877 (13.08)	75 (5.15)

Knows HIV status							
No	390 (6.24)	666 (5.3)	682 (9.39)	665 (3.77)	337 (3.25)	2989 (4.83)	170 (2.26)
Yes	52 (6.32)	1049 (4.36)	791 (6.48)	1175 (2.92)	281 (1.78)	772 (3.74)	498 (2.06)

Heard about AIDS							
No	3 (4.08)	2 (1.46)	69 (11.11)	4 (0.66)	1 (0.45)	122 (1.84)	0 (0.00)
Yes	443 (6.24)	1719 (4.68)	1480 (7.57)	1835 (3.19)	622 (2.37)	3700 (4.73)	668 (2.10)

Comprehensive knowledge							
No	321 (6.13)	880 (4.52)	1139 (8.55)	1005 (2.89)	420 (2.79)	2684 (4.35)	255 (2.11)
Yes	124 (6.49)	841 (4.84)	411 (5.99)	834 (3.58)	202 (1.79)	1138 (4.90)	412 (2.09)

Reads newspaper/magazine							
No	245 (4.92)	712 (3.83)	1124 (8.29)	1098 (2.67)	335 (3.17)	2393 (3.89)	483 (2.02)
Yes	200 (9.18)	1008 (5.53)	426 (6.44)	740 (4.36)	282 (1.79)	1404 (6.13)	183 (2.35)

Listens to radio							
No	16 (2.93)	129 (3.17)	465 (8.74)	342 (2.02)	73 (2.13)	621 (2.71)	54 (1.40)
Yes	428 (6.48)	1591 (4.85)	1085 (7.3)	1495 (3.63)	549 (2.40)	3187 (5.17)	613 (2.20)

Watches television							
No	132 (5.05)	566 (3.63)	1025 (8.04)	1101 (2.57)	282 (2.38)	1594 (4.03)	390 (1.83)
Yes	313 (6.90)	1155 (5.44)	525 (7.07)	737 (4.85)	340 (2.34)	2212 (4.91)	277 (2.64)

Variable							
Country	Uganda	Zambia	Zimbabwe				
Age (mean ± SD)	32.21 (9.65)	32.88 (10.41)	32.26 (9.07)				

Age group							
15–19	181 (4.86)	313 (6.8)	92 (3.20)				
20–24	369 (5.13)	425 (5.77)	315 (4.71)				
25–29	395 (5.87)	528 (6.63)	397 (5.55)				
30–34	409 (7.25)	536 (7.64)	365 (5.71)				
35–39	300 (6.94)	465 (8.41)	279 (5.78)				
40–44	249 (7.57)	348 (8.53)	185 (5.23)				
45–49	162 (7.33)	219 (7.61)	136 (5.49)				
50–54	118 (24.75)	152 (14.72)	91 (9.47)				
55–59		91 (12.41)					

Sex							
Male	1639 (22.01)	2699 (15.13)	1705 (10.9)				
Female	544 (2.08)	377 (1.61)	155 (0.80)				

Religion							
Christian	1697 (6.36)	2965 (7.36)	1255 (4.43)				
Islam	363 (8.39)	22 (8.68)	21 (8.91)				
Traditional	Na	Na	120 (10.21)				
No religion	Na	13 (12.48)	464 (9.00)				
Others	21 (5.8)	2 (6.96)	1 (2.89)				

Marital status							
Never married	247 (6.18)	624 (8.94)	200 (4.44)				
Married	1123 (6.86)	2252 (7.21)	1526 (5.67)				
Cohabiting	606 (5.89)	25 (7.62)	53 (5.37)				
Widowed	23 (5.18)	19 (3.5)	11 (1.79)				
Divorced	5 (2.94)	105 (7.35)	36 (3.76)				
Separated	178 (7.81)	52 (7.45)	34 (3.74)				

Educational level							
No education	130 (3.28)	159 (4.70)	28 (3.89)				
Primary	1375 (6.99)	1555 (7.47)	494 (4.92)				
Secondary	508 (6.90)	1199 (8.34)	1182 (5.47)				
Higher	170 (6.58)	163 (6.26)	156 (6.09)				

Employment status							
Not working	142 (2.51)	516 (3.88)	433 (2.88)				
Working	2041 (7.32)	2556 (9.19)	1427 (7.18)				

Wealth index							
Poorest	337 (5.69)	505 (8.23)	341 (5.56)				
Poorer	401 (6.27)	525 (8.24)	325 (5.27)				
Middle	447 (6.96)	593 (8.91)	336 (5.49)				
Richer	516 (7.65)	580 (7.82)	461 (5.45)				
Richest	482 (5.96)	418 (5.84)	397 (4.94)				

Residence							
Rural	1749 (6.66)	2035 (8.32)	1165 (5.31)				
Urban	434 (5.92)	1041 (6.22)	695 (5.35)				

Resides with spouse/partner							
No	167 (4.63)	87 (5.05)	179 (3.54)				
Yes	1565 (6.77)	1923 (6.83)	1371 (6.14)				

First sex							
Above 18 years	836 (6.12)	1166 (5.63)	1080 (5.05)				
Below 18 years	1346 (6.76)	1909 (9.33)	762 (5.77)				

Circumcision							
No	996 (21.25)	1955 (14.91)	1486 (10.77)				
Yes	646 (23.31)	426 (13.9)	218 (11.83)				

Knows HIV status							
No	708 (8.13)	1435 (8.12)	878 (6.16)				
Yes	1475 (5.93)	1637 (6.99)	981 (4.79)				

Heard about AIDS							
No	2 (3.36)	12 (7.15)	6 (1.68)				
Yes	2181 (6.51)	3065 (7.47)	1854 (5.36)				

Comprehensive knowledge							
No	1155 (6.42)	1923 (7.22)	821 (5.17)				
Yes	1028 (6.60)	1154 (7.92)	1039 (5.46)				

Reads newspaper/magazine							
No	1422 (5.77)	1691 (6.61)	719 (4.17)				
Yes	756 (8.49)	1379 (8.88)	1141 (6.46)				

Listens to radio							
No	244 (4.04)	644 (5.44)	473 (3.87)				
Yes	1939 (7.04)	2428 (8.28)	1386 (6.12)				

Watches television							
No	1227 (5.26)	1575 (6.76)	757 (4.38)				
Yes	956 (9.31)	1501 (8.40)	1103 (6.26)				

Na, not available. Frequency and percentages are survey weighted.

**Table 3:** Bivariate analysis of higher-risk sexual behavior with country.

Variable	Crude hazard ratio (cHR)
Country	cHR (95% CI)

Rwanda	1.00 (reference)
Ghana	3.72 (3.37–4.10)^*∗∗∗*^
Kenya	2.14 (1.93–2.37)^*∗∗∗*^
Lesotho	4.26 (3.86–4.69)^*∗∗∗*^
Malawi	1.99 (1.80–2.19)^*∗∗∗*^
Nigeria	2.78 (2.55–3.03)^*∗∗∗*^
Namibia	1.37 (1.22–1.55)^*∗∗∗*^
Uganda	3.98 (3.63–4.36)^*∗∗∗*^
Zambia	4.66 (4.26–5.09)^*∗∗∗*^
Zimbabwe	3.03 (2.75–3.32)^*∗∗∗*^

^*∗*^*P* < 0.05. ^*∗∗*^*P* < 0.01. ^*∗∗∗*^*P* < 0.001. CI, confidence interval.

**Table 4:** Adjusted hazard ratio of respondents practicing H-RSB from country specific pooled dataset.

Country	Ghana	Kenya	Lesotho	Malawi	Namibia	Nigeria	Rwanda
Variables	Adjusted hazard ratio, aHR (95% CI)	Adjusted hazard ratio, aHR (95% CI)	Adjusted hazard ratio, aHR (95% CI)	Adjusted hazard ratio, aHR (95% CI)	Adjusted hazard ratio, aHR (95% CI)	Adjusted hazard ratio, aHR (95% CI)	Adjusted hazard ratio, aHR (95% CI)

Age group							
15–19	1.00 (reference)	1.00 (reference)	1.00 (reference)	1.00 (reference)	1.00 (reference)	1.00 (reference)	1.00 (reference)
20–24	0.46 (0.22–1.00)^*∗*^	1.30 (0.96–1.77)	1.41 (0.93–2.15)	0.67 (0.44–1.01)	2.07 (1.41–3.04)^*∗∗∗*^	1.05 (0.6–1.85)	0.32 (0.1–0.96)^*∗*^
25–29	0.56 (0.28–1.12)	1.20 (0.84–1.7)	2.04 (1.36–3.07)^*∗∗*^	0.64 (0.43–0.96)^*∗*^	2.02 (1.35–3.02)^*∗∗*^	1.10 (0.65–1.89)	0.41 (0.14–1.2)
30–34	0.52 (0.26–1.05)	1.26 (0.88–1.81)	2.48 (1.65–3.74)^*∗∗∗*^	0.84 (0.57–1.26)	1.78 (1.14–2.76)^*∗*^	1.40 (0.82–2.38)	0.56 (0.19–1.63)
35–39	0.53 (0.26–1.06)	1.29 (0.89–1.87)	2.19 (1.44–3.33)^*∗∗∗*^	0.68 (0.45–1.02)	1.46 (0.9–2.36)	1.73 (1.02–2.94)^*∗*^	0.47 (0.16–1.4)
40–44	0.56 (0.28–1.12)	1.04 (0.72–1.51)	2.15 (1.41–3.28)^*∗∗∗*^	0.73 (0.48–1.11)	1.48 (0.89–2.45)	2.00 (1.18–3.4)^*∗*^	0.48 (0.16–1.43)
45–49	0.56 (0.27–1.12)	1.13 (0.77–1.67)	2.1 (1.36–3.26)^*∗∗*^	0.82 (0.53–1.27)	1.40 (0.82–2.36)	2.16 (1.27–3.65)^*∗∗*^	0.59 (0.19–1.77)
50–54	0.66 (0.32–1.35)	1.20 (0.81–1.79)	1.30 (0.71–2.38)	0.61 (0.38–0.99)^*∗*^	2.28 (1.21–4.31)^*∗*^	1.92 (1.1–3.36)^*∗*^	0.58 (0.19–1.75)
55–59	0.60 (0.29–1.25)		1.41 (0.76–2.64)		1.25 (0.43–3.65)	2.30 (1.31–4.02)^*∗∗*^	0.63 (0.21–1.94)
60–64					2.65 (0.6–11.64)		

Sex							
Male	1.00 (reference)	1.00 (reference)	1.00 (reference)	1.00 (reference)	1.00 (reference)	1.00 (reference)	1.00 (reference)
Female	0.01 (0–0.04)^*∗∗∗*^	0.21 (0.16–0.27)^*∗∗∗*^	0.42 (0.32–0.55)^*∗∗∗*^	0.04 (0.01–0.22)^*∗∗∗*^	0.20 (0.14–0.28)^*∗∗∗*^	0.05 (0.03–0.08)^*∗∗∗*^	0.06 (0.05–0.09)^*∗∗∗*^

Religion							
Christians	1.00 (reference)	1.00 (reference)	1.00 (reference)	1.00 (reference)	1.00 (reference)	1.00 (reference)	1.00 (reference)
Islam	0.85 (0.67–1.08)	1.52 (1.22–1.91)^*∗∗∗*^	0.32 (0.06–1.62)	1.41 (1.13–1.75)^*∗∗*^		0.90 (0.65–1.24)	1.24 (0.68–2.23)
Traditional	1.16 (0.93–1.44)		1.02 (0.7–1.51)	1.59 (1.12–2.24)^*∗∗*^		3.25 (0.45–23.33)	1.15 (0.42–3.16)
No religion	0 (0–0)^*∗∗∗*^	1.81 (1.45–2.24)^*∗∗∗*^	1.54 (0.9–2.63)		0.74 (0.51–1.06)		2.05 (1.03–4.05)^*∗*^
Other religion	1.62 (1.2–2.2)^*∗∗*^				1.75 (0.75–4.06)		0.95 (0.22–4.17)

Marital status							
Never married		1.00 (reference)			1.00 (reference)		
Married	1.00 (reference)	2.33 (1.81–2.98)^*∗∗∗*^	1.00 (reference)	1.00 (reference)	1.46 (1.1–1.95)^*∗∗*^	1.00 (reference)	1.00 (reference)
Cohabiting	1.46 (1.16–1.83)^*∗∗*^	2.71 (1.79–4.09)^*∗∗∗*^	1.36 (0.9–2.06)	0.94 (0.75–1.18)	1.73 (1.31–2.29)^*∗∗∗*^	1.13 (0.89–1.44)	2.18 (1.76–2.69)^*∗∗∗*^
Widowed		1.77 (1.04–3)^*∗*^			0.44 (0.12–1.62)		
Divorced		1.41 (0.85–2.34)			1.76 (0.63–4.89)		
Separated		2.71 (1.91–3.83)^*∗∗∗*^			2.13 (1.36–3.34)^*∗∗*^		

Educational level							
No education	1.00 (reference)	1.00 (reference)	1.00 (reference)	1.00 (reference)	1.00 (reference)	1.00 (reference)	1.00 (reference)
Primary	1.17 (0.89–1.53)	0.85 (0.68–1.06)	1.03 (0.8–1.32)	1.11 (0.88–1.42)	0.80 (0.6–1.08)	0.96 (0.86–1.09)	1.07 (0.82–1.41)
Secondary	1.08 (0.84–1.4)	0.74 (0.57–0.97)^*∗*^	0.92 (0.69–1.24)	0.89 (0.66–1.21)	0.62 (0.45–0.86)^*∗∗*^	0.96 (0.84–1.1)	0.84 (0.53–1.32)
Higher	0.73 (0.49–1.1)	0.81 (0.59–1.12)	0.76 (0.47–1.23)	0.67 (0.36–1.25)	0.79 (0.49–1.27)	0.70 (0.58–0.86)^*∗∗*^	0.68 (0.27–1.72)

Employment status							
Not working	1.00 (reference)	1.00 (reference)	1.00 (reference)	1.00 (reference)	1.00 (reference)	1.00 (reference)	1.00 (reference)
Working	0.98 (0.6–1.59)	1.51 (1.2–1.9)^*∗∗∗*^	1.32 (1.15–1.52)^*∗∗∗*^	1.17 (0.92–1.48)	1.29 (1.04–1.61)^*∗*^	1.12 (0.93–1.34)	1.05 (0.62–1.77)

Wealth index							
Poorest	1.00 (reference)	1.00 (reference)	1.00 (reference)	1.00 (reference)	—	1.00 (reference)	1.00 (reference)
Poorer	1.02 (0.78–1.33)	0.82 (0.68–0.99)^*∗*^	0.91 (0.75–1.1)	1.06 (0.85–1.31)		0.92 (0.82–1.03)	1.42 (1.02–1.98)^*∗*^
Middle	1.18 (0.87–1.59)	0.84 (0.7–1.02)	0.71 (0.57–0.87)^*∗∗*^	1.02 (0.83–1.26)		0.76 (0.66–0.87)^*∗∗∗*^	1.35 (0.97–1.88)
Richer	1.29 (0.91–1.82)	0.87 (0.7–1.06)	0.64 (0.51–0.81)^*∗∗∗*^	1.08 (0.86–1.35)		0.73 (0.62–0.86)^*∗∗∗*^	1.14 (0.81–1.61)
Richest	1.62 (1.11–2.36)^*∗*^	0.92 (0.72–1.17)	0.62 (0.46–0.85)^*∗∗*^	1.09 (0.85–1.41)		0.65 (0.53–0.79)^*∗∗∗*^	1.25 (0.84–1.86)

Residence							
Rural	1.00 (reference)	1.00 (reference)	1.00 (reference)	1.00 (reference)	1.00 (reference)	1.00 (reference)	1.00 (reference)
Urban	0.79 (0.63–1)	1.15 (0.99–1.34)	0.88 (0.71–1.09)	0.75 (0.58–0.96)^*∗*^	1.02 (0.82–1.28)	0.80 (0.72–0.90)^*∗∗∗*^	1.03 (0.76–1.4)

Resides with spouse/partner							
No	1.00 (reference)	—	1.00 (reference)	1.00 (reference)	—	1.00 (reference)	1.00 (reference)
Yes	0.9 (0.73–1.11)	—	0.79 (0.69–0.9)^*∗∗∗*^	1.08 (0.81–1.43)		1.19 (1.01–1.41)^*∗*^	0.76 (0.5–1.15)

First sex							
Above 18 years	1.00 (reference)	1.00 (reference)	1.00 (reference)	1.00 (reference)	1.00 (reference)	1.00 (reference)	1.00 (reference)
Below 18 years	1.74 (1.47–2.06)^*∗∗∗*^	2.15 (1.85–2.50)^*∗∗∗*^	1.50 (1.31–1.72)^*∗∗∗*^	1.46 (1.27–1.68)^*∗∗∗*^	1.99 (1.62–2.44)^*∗∗∗*^	1.55 (1.41–1.70)^*∗∗∗*^	2.13 (1.69–2.67)^*∗∗∗*^

Circumcision							
No	1.00 (reference)	1.00 (reference)	1.00 (reference)	1.00 (reference)	1.00 (reference)	1.00 (reference)	—
Yes	1.2 (0.84–1.71)	0.72 (0.58–0.91)^*∗∗*^	1.21 (0.91–1.59)	1.04 (0.86–1.26)	1.26 (0.89–1.79)	1.23 (0.70–2.16)	

Knows HIV status							
No	1.00 (reference)	1.00 (reference)	1.00 (reference)	1.00 (reference)	—	1.00 (reference)	1.00 (reference)
Yes	1.12 (0.92–1.37)	1.08 (0.93–1.25)	0.93 (0.79–1.1)	1.01 (0.84–1.2)		0.86 (0.76–0.97)^*∗*^	1.05 (0.72–1.52)

Heard about AIDS							
No	1.00 (reference)	—	1.00 (reference)	1.00 (reference)	1.00 (reference)	1.00 (reference)	—
Yes	1.87 (0.55–6.35)	—	3.89 (0.58–26.11)	1.74 (0.58–5.19)	5.29 (0.75–37.45)	1.35 (1.04–1.75)^*∗*^	

Comprehensive knowledge							
No	1.00 (reference)	1.00 (reference)	1.00 (reference)	1.00 (reference)	1.00 (reference)	1.00 (reference)	1.00 (reference)
Yes	0.96 (0.8–1.16)	1.07 (0.92–1.24)	0.90 (0.77–1.06)	1.12 (0.98–1.29)	1.21 (0.94–1.57)	0.96 (0.88–1.05)	1.04 (0.83–1.3)

Reads newspaper/magazine							
No	1.00 (reference)	1.00 (reference)	1.00 (reference)	1.00 (reference)	1.00 (reference)	1.00 (reference)	1.00 (reference)
Yes	1 (0.81–1.24)	1.01 (0.88–1.16)	0.99 (0.84–1.18)	1.15 (0.98–1.34)	0.74 (0.58–0.94)^*∗*^	1.08 (0.96–1.21)	1.09 (0.85–1.39)

Listens to radio							
No	1.00 (reference)	1.00 (reference)	1.00 (reference)	1.00 (reference)	1.00 (reference)	1.00 (reference)	1.00 (reference)
Yes	0.92 (0.63–1.35)	1.05 (0.81–1.35)	1.05 (0.89–1.23)	1.14 (0.94–1.37)	1.09 (0.80–1.48)	1.01 (0.90–1.14)	1.05 (0.69–1.6)

Watches television							
No	1.00 (reference)	1.00 (reference)	1.00 (reference)	1.00 (reference)	1.00 (reference)	1.00 (reference)	1.00 (reference)
Yes	1.06 (0.85–1.32)	0.99 (0.86–1.15)	1.15 (0.96–1.38)	1.15 (0.99–1.34)	1.01 (0.79–1.29)	1.12 (0.99–1.25)	1.08 (0.86–1.35)

Period							
One	1.00 (reference)	1.00 (reference)	1.00 (reference)	1.00 (reference)	1.00 (reference)	1.00 (reference)	1.00 (reference)
Two	0.50 (0.24–1.10)	0.50 (0.33–0.77)^*∗∗∗*^	0.58 (0.47–0.72)^*∗∗∗*^	0.92 (0.60–1.41)	0.54 (0.37–0.80)^*∗∗*^	0.84 (0.47–1.53)	1.51 (0.74–3.09)
Three	0.71 (0.34–1.51)	0.51 (0.33–0.80)^*∗∗∗*^	0.54 (0.43–0.68)^*∗∗∗*^	1.39 (0.90–2.14)	0.56 (0.36–0.87)^*∗∗*^	1.48 (0.81–2.67)	1.48 (0.72–3.06)

Country	Uganda	Zambia	Zimbabwe				
Variables	Adjusted hazard ratio, aHR (95% CI)	Adjusted hazard ratio, aHR (95% CI)	Adjusted hazard ratio, aHR (95% CI)				
Age group							
15–19	1.00 (reference)	1.00 (reference)	1.00 (reference)				
20–24	0.64 (0.45–0.92)^*∗*^	0.76 (0.48–1.22)	0.8 (0.48–1.35)				
25–29	0.67 (0.48–0.95)^*∗*^	0.91 (0.58–1.44)	0.66 (0.40–1.10)				
30–34	0.72 (0.51–1.02)	0.89 (0.57–1.41)	0.61 (0.37–1.02)				
35–39	0.71 (0.5–1.01)	0.96 (0.61–1.52)	0.55 (0.33–0.91)^*∗*^				
40–44	0.72 (0.5–1.02)	0.89 (0.56–1.42)	0.47 (0.28–0.78)^*∗∗*^				
45–49	0.69 (0.48–1)^*∗*^	0.82 (0.51–1.31)	0.49 (0.29–0.83)^*∗∗*^				
50–54	0.73 (0.5–1.07)	0.88 (0.54–1.42)	0.45 (0.26–0.78)^*∗∗*^				
55–59		0.76 (0.46–1.25)					

Sex							
Male	1.00 (reference)	1.00 (reference)	1.00 (reference)				
Female	0.04 (0.01–0.14)^*∗∗∗*^	0.06 (0.05–0.08)^*∗∗∗*^	0.04 (0.01–0.10)^*∗∗∗*^				

Religion							
Christians	1.00 (reference)	—	1.00 (reference)				
Islam	1.46 (1.25–1.7)^*∗∗∗*^		1.42 (0.85–2.37)				
Traditional			1.29 (1.05–1.58)^*∗*^				
No religion			1.16 (1.02–1.32)^*∗*^				
Other religion	1.01 (0.63–1.6)		0.42 (0.1–1.71)				

Marital status							
Never married							
Married	1.00 (reference)	1.00 (reference)	1.00 (reference)				
Cohabiting	1.25 (1.12–1.39)^*∗∗∗*^	1.10 (0.7–1.73)	1.25 (0.91–1.71)				
Widowed							
Divorced							
Separated							

Educational level							
No education	1.00 (reference)	1.00 (reference)	1.00 (reference)				
Primary	1.34 (1.08–1.68)^*∗∗*^	1.16 (0.95–1.42)	0.73 (0.48–1.11)				
Secondary	1.23 (0.96–1.57)	1.17 (0.94–1.46)	0.74 (0.48–1.13)				
Higher	1.02 (0.76–1.38)	0.74 (0.54–1)	0.71 (0.44–1.14)				

Employment status							
Not working	1.00 (reference)	1.00 (reference)	1.00 (reference)				
Working	1.07 (0.82–1.39)	0.98 (0.84–1.15)	1.19 (1.03–1.37)^*∗*^				

Wealth index							
Poorest	1.00 (reference)	—	1.00 (reference)				
Poorer	1.03 (0.88–1.2)		0.98 (0.83–1.16)				
Middle	1.15 (0.98–1.35)		1.01 (0.85–1.21)				
Richer	1.17 (0.99–1.37)		0.83 (0.67–1.03)				
Richest	1.02 (0.84–1.24)		0.75 (0.57–0.99)^*∗*^				

Residence							
Rural	1.00 (reference)	1.00 (reference)	1.00 (reference)				
Urban	0.83 (0.71–0.97)^*∗*^	0.67 (0.59–0.77)^*∗∗∗*^	1.09 (0.89–1.33)				
Resides with spouse/partner							
No	1.00 (reference)	1.00 (reference)	1.00 (reference)				
Yes	1.16 (0.97–1.4)	0.86 (0.67–1.1)	1.05 (0.89–1.25)				

First sex							
Above 18 years	1.00 (reference)	1.00 (reference)	1.00 (reference)				
Below 18 years	1.52 (1.38–1.67)^*∗∗∗*^	1.48 (1.34–1.63)^*∗∗∗*^	1.80 (1.61–2.01)^*∗∗∗*^				

Circumcision							
No	1.00 (reference)	—	1.00 (reference)				
Yes	0.94 (0.83–1.07)		1.12 (0.94–1.33)				

Knows HIV status							
No	1.00 (reference)	1.00 (reference)	1.00 (reference)				
Yes	1 (0.89–1.13)	0.98 (0.88–1.1)	0.85 (0.75–0.97)^*∗*^				

Heard about AIDS							
No	1.00 (reference)	1.00 (reference)	1.00 (reference)				
Yes	1.5 (0.41–5.43)	0.61 (0.16–2.4)	1.00 (0.42–2.39)				

Comprehensive knowledge							
No	1.00 (reference)	1.00 (reference)	1.00 (reference)				
Yes	0.93 (0.84–1.03)	1.05 (0.95–1.16)	1.08 (0.97–1.21)				

Reads newspaper/magazine							
No	1.00 (reference)	1.00 (reference)	1.00 (reference)				
Yes	1.05 (0.93–1.17)	1.14 (1.02–1.29)^*∗*^	1.17 (1.03–1.33)^*∗*^				

Listens to radio							
No	1.00 (reference)	1.00 (reference)	1.00 (reference)				
Yes	1.06 (0.88–1.28)	0.93 (0.82–1.05)	1.05 (0.92–1.20)				

Watches television							
No	1.00 (reference)	1.00 (reference)	1.00 (reference)				
Yes	1.24 (1.1–1.38)^*∗∗∗*^	1.21 (1.08–1.35)^*∗∗*^	1.14 (1.00–1.29)				

Period							
One	1.00 (reference)	1.00 (reference)	1.00 (reference)				
Two	0.96 (0.83–1.12)	0.60 (0.42–0.86)^*∗∗*^	1.29 (1.11– 1.50)^*∗∗*^				
Three	1.04 (0.91–1.20)	0.65 (0.45–0.95)^*∗*^	1.73 (1.48– 2.02)^*∗∗∗*^				

^*∗*^*P* < 0.05. ^*∗∗*^*P* < 0.01. ^*∗∗∗*^*P* < 0.001. CI, confidence interval; *P*, probability value. *P* values were calculated using Cox proportional hazard with constant time of weighted data.

## Data Availability

An application requesting for the use of the Demographic and Health Survey's data was sent to the DHS website. Data were then used after approval was obtained. The datasets generated and/or analyzed during the current study are publicly available in the Demographic and Health Survey Repository, http://dhsprogram.com/data/available-datasets.cfm.
